# Dirac-Rashba fermions and quantum valley Hall insulators in graphene-based 2D heterostructures

**DOI:** 10.1016/j.isci.2025.112818

**Published:** 2025-06-04

**Authors:** Bo-Wen Yu, Bang-Gui Liu

**Affiliations:** 1 Beijing National Laboratory for Condensed Matter Physics, Institute of Physics, Chinese Academy of Sciences, Beijing 100190, China; 2 School of Physical Sciences, University of Chinese Academy of Sciences, Beijing 100049, China

**Keywords:** Chemistry, Physics, Materials science

## Abstract

For promising Dirac electronic properties and functionalities, we study five 2D heterostructures consisting of graphene and monolayer transition metal dichalcogenides by means of first-principles investigation and an effective low-energy model. It is revealed from first-principles bands that the Dirac energy bands are gapped (0.1 ∼ 0.5 meV) and their relativistic dispersions are robust up to the energy window of 0.4 eV at least. The model parameters are determined by fitting the first-principles calculated bands in each of the heterostructures. It is shown that Dirac-Rashba fermions are hosted in the ${\text{WSe}}_{2}$/graphene and ${\text{MoSe}}_{2}$/graphene/WSe_2_, and quantum valley Hall insulators can be achieved in all these heterostructures. Further analysis elucidates features of Berry curvature distribution and interactions of the orbitals and spins. These can be useful in future exploration for more effects and functionalities in 2D heterostructures.

## Highlights

• Dirac-Rashba fermion systems are proposed from graphene-based heterostructures
• Quantum valley Hall insulator is elucidated for these heterostructures
• There are wide energy windows for relativistic energy dispersion in the bands
• The effective model is determined by reproducing DFT calculated results

## Graphical abstract

**Figure:**
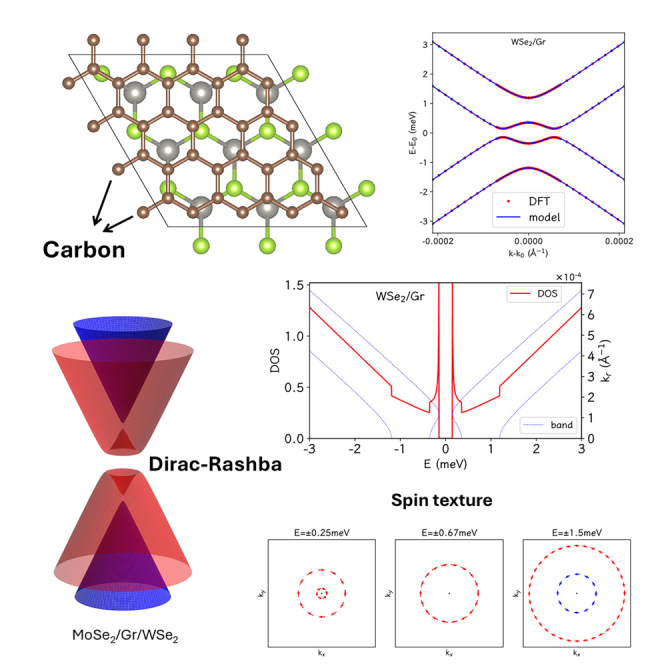

## Introduction

Since the advent of graphene in 2004,^1^^,^^2^^,^^3^ two-dimensional (2D) materials have been extensively explored for their important phenomena and practical applications. Transition metal dichalcogenides (TMDs) as 2D semiconductors are well known for their unique qualities in scientific research and engineering applications.^4^^,^^5^^,^^6^ It is known that a combination of these two kinds of famous 2D materials can lead to much more rich and amazing properties. Actually, there have been many experimental achievements of heterostructures of graphene and monolayer TMDs, thanks to the great development of experimental technology and methods.^7^^,^^8^^,^^9^^,^^10^^,^^11^^,^^12^^,^^13^^,^^14^^,^^15^^,^^16^^,^^17^^,^^18^^,^^19^^,^^20^^,^^21^^,^^22^

The graphene is the ideal 2D material for studying the Dirac fermions and topological properties in two dimensions because of its multiple degrees of freedom. Its orbital, spin, and valley features can be combined to form various interesting properties and topological structures.^2^^,^^23^^,^^24^^,^^25^^,^^26^^,^^27^^,^^28^ However the tiny spin-orbit coupling (SOC) in pure graphene causes some limitations in various aspects. It is believed that some TMDs can be added to form graphene-based 2D heterostructures with strong SOC. As for important effect of SOC, Rashba effect near band edges of semiconductors is vital to determining carrier properties and electronic functionalities in semiconductor technology.^29^^,^^30^^,^^31^^,^^32^^,^^33^ Considering recent experimental advances,^2^^,^^25^^,^^26^^,^^31^^,^^32^^,^^33^^,^^34^^,^^35^^,^^36^ it is highly desirable to explore new phenomena and novel effects induced by the Dirac and Rashba features.

Here, we investigate 2D heterostructures consisting of graphene and monolayer TMDs (${\text{MoSe}}_{2}$ and ${\text{WSe}}_{2}$) by means of first-principles methods and effective k·p low-energy models. These two TMDs are chosen in order to align the Dirac cones of graphene with their semiconductor gaps. We study the electronic structures of their optimized structures and construct a unified effectively low-energy model for further investigation. The parameters of the model are determined by fitting with the first-principles bands. It is found that the linear band dispersion is kept in a wide energy window and there are some modifications in the bands, which leads to Dirac-Rashba fermions in the heterostructures. We also calculate their Berry curvatures and find that the effective model hosts quantum valley Hall effect for some of the heterostructures. The detailed data and further analyses will be presented below.

## Results and discussion

### Structures of graphene-based heterostructures

**Figure 1:**
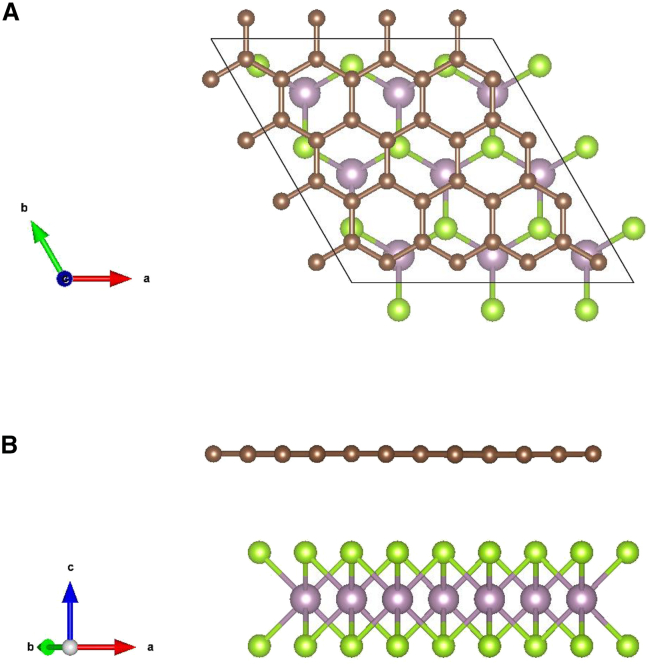
The structure of heterostructures The top-view (A) and side-view (B) structure of ${\text{MoSe}}_{2}$/graphene (${\text{WSe}}_{2}$/graphene) heterostructure. Carbon, Mo (W), and Se atoms are brown, pink, and green, respectively. The primitive vectors are indicated with the arrows.

The graphene and 2D TMDs structures have been extensively studied experimentally and theoretically for their interesting physical properties and applications.^21^^,^^22^^,^^26^^,^^36^^,^^37^^,^^38^^,^^39^^,^^40^^,^^41^^,^^42^^,^^43^^,^^44^^,^^45^^,^^46^^,^^47^^,^^48^^,^^49^^,^^50^^,^^51^ It was shown that the moiré pattern of a heterostructure can be eliminated by annealing process at a high temperature,^22^ which means that high stability can be achieved in some heterostructures without moiré pattern. We can construct some 2D heterostructures by combining graphene and appropriate H-phase TMD monolayers, in order to achieve interesting 2D electronic structures and topological features. It is expected that while the Dirac cones of graphene will be changed by TMD monolayers, the main important features of graphene will be kept. We choose MoSe2 and WSe2 as TMD monolayers because the Dirac points (modified) are located approximately in the middle of their semiconductor gaps. Consequently, we can realize ${\text{MoSe}}_{2}$/graphene, ${\text{WSe}}_{2}$/graphene, and ${\text{MoSe}}_{2}$/graphene/WSe_2_ as our 2D heterostructures. The two bilayer heterostructures are described in Figure 1. We also investigate structural variants of ${\text{MoSe}}_{2}$/graphene and ${\text{WSe}}_{2}$/graphene to show that the electronic structure and main properties are not sensitive to variation of inter-layer structure. The structural variants are shown in Figure S2 (supplemental information), and the trilayer heterostructures are shown in Figure S3. In addition, we construct two symmetrical trilayer heterostructures for comparison: ${\text{WSe}}_{2}$/graphene/WSe_2_ and ${\text{MoSe}}_{2}$/graphene/MoSe_2_.

The computational models contain one monolayer graphene and one or two monolayers of TMDs. It is necessary to reconcile the different lattice lengths of 4×4 graphene cells and 3×3 TMD cells. Graphene has bond length a=1.42 Å and lattice constant $a_{1}=a_{2}=2.46$ Å^2^. Both ${\text{MoSe}}_{2}$ and ${\text{WSe}}_{2}$ have $a_{1}^{\prime }=a_{2}^{\prime }=3.30$ Å,^37^ and then $3a_{1}^{\prime }/\left(4a_{1}\right)=1.006$. We apply a biaxial strain 0.6% to graphene to remove the small mismatch. Such a small strain can cause only a tiny change in the electronic structure of graphene.^25^ It is clear that there is no twisting in these 2D heterostructures. In order to effectively perform structural optimization, it is helpful to choose a little smaller initial value for the inter-layer distance between graphene monolayer and TMD monolayer. The optimized distance value is 3.48 Å for ${\text{MoSe}}_{2}$/graphene, 3.51 Å for ${\text{WSe}}_{2}$/graphene, 3.46 Å for ${\text{MoSe}}_{2}$/graphene/WSe_2_, 3.42 Å for ${\text{WSe}}_{2}$/graphene/WSe_2_, or 3.44 Å for ${\text{MoSe}}_{2}$/graphene/MoSe_2_.

### Electronic energy bands

**Figure 2:**
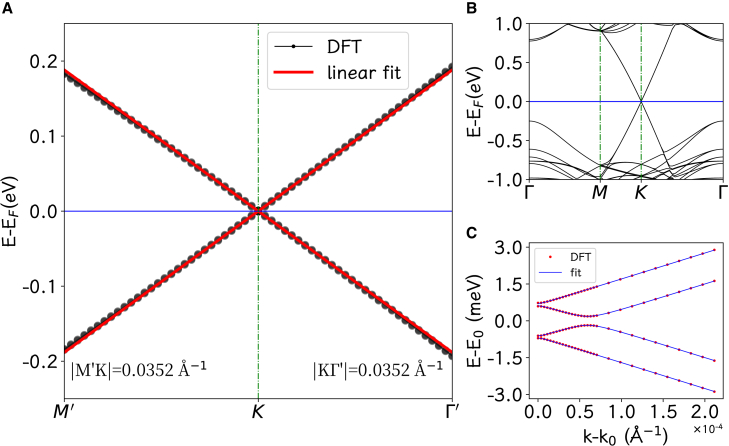
The electronic band structure of representative heterostructure The electronic band structure of ${\text{MoSe}}_{2}$/graphene/WSe_2_ heterostructure. (A) The energy bands near the K point to show the graphene-like linear dispersion between −0.25 and 0.25 eV, where the black lines describe the results of DFT calculation and the red lines the linear fitting. (B) The band structure of the 2D heterostructure in a wide energy window between −1 and 1 eV, showing the band features from the graphene and the 2D TMDs. (C) The band structure between −3 and 3 meV, demonstrating the Rashba effect near the Fermi level, where the red points are from DFT calculation and the blue lines the result of the fitting of the bands of the low energy model. The horizontal axis is the distance from the K point in the Brillouin zone.

With the optimized structures, we can investigate their electronic structures. The band structure of ${\text{MoSe}}_{2}$/graphene/WSe_2_ heterostructure is presented in Figure 2. It is very interesting that the Dirac cone of graphene is located within the semiconductor gaps of monolayer ${\text{MoSe}}_{2}$ and ${\text{WSe}}_{2}$ and there is an ideal linear dispersion in the graphene-dominant bands within the energy window [−0.2 eV, 0.2 eV]. This energy window corresponds to a circle of radius of 0.035 Å^−1^ around K (K′) point in the Brillouin zone. Actually, the bands near the Dirac cone are modified by the TMD monolayers, as shown in Figure 2C. The modification is limited to the small region near K (K′) point. The conduction band minimum (CBM) and the valence band maximum (VBM) are both moved a little bit away from K (K′) point, forming a circle around K (K′) point. Usually, such a band edge indicates some Rashba effect.^29^^,^^30^^,^^31^^,^^33^ The energy window of the band modification is between −1.5 and 1.5 meV. The band structures of other heterostructures are shown in Figure S4. ${\text{WSe}}_{2}$/graphene and its variants have similar band structures. The ${\text{MoSe}}_{2}$/graphene and the two symmetric trilayer heterostructures have overall similar band structures, but their CBB and VBT are both at K (K′) point.

### Effective low-energy Hamiltonian

As shown in Figures 2 and S4, the whole band structures are complex, but the low-energy parts of the band structures are simple, featuring the Dirac cones and linear dispersions. It is clear that the low-energy parts are similar to the low-energy bands of monolayer graphene. In each case, there are four bands, two conduction bands and two valence bands, for the low-energy part, and there is a symmetry between the conduction bands and the valence bands, as clearly revealed in Figure S5. Therefore, we use the following k·p Hamiltonian (1) to describe the low-energy bands near K (K′) point in all the cases.

(Equation 1) $$\begin{array}{cc} \hat{H}= & v_{F}\left({\hat{\sigma }}_{x}k_{x}+{\hat{\tau }}_{z}{\hat{\sigma }}_{y}k_{y}\right)+\frac{R}{2}\left({\hat{\sigma }}_{y}{\hat{s}}_{x}-{\hat{\tau }}_{z}{\hat{\sigma }}_{x}{\hat{s}}_{y}\right)\\ & +\frac{A+B}{2}{\hat{\sigma }}_{z}+\frac{A-B}{2}{\hat{\tau }}_{z}{\hat{s}}_{z}\text{,} \end{array}$$

where $\vec{k}=\left(k_{x},k_{y}\right)$ describes the k-vector in the 2D Brillouin zone, $\hat{\vec{\sigma }}=\left({\hat{\sigma }}_{x},{\hat{\sigma }}_{y},{\hat{\sigma }}_{z}\right)$ are Pauli matrixes for the sublattice orbitals, $\left({\hat{s}}_{x},{\hat{s}}_{y},{\hat{s}}_{z}\right)$ are Pauli matrixes describing spin, and ${\hat{\tau }}_{z}$ is used to describe the valleys (with eigenvalue 1 for K and −1 for K′). $\hat{\vec{\sigma }}$ can be considered to describe a pseudo-spin. The first term describes the low-energy bands of pure graphene, the second term is the relativistic spin-orbit effect due to the TMD monolayers, and the last two terms are used to cause energy gaps due to the interaction between graphene and TMD monolayers.^26^ Here, it should be noted that we have hidden the unit matrixes corresponding to ${\hat{\sigma }}_{0}$ and ${\hat{s}}_{0}$ in Hamiltonian (1), as is usually done.^26^ It can be seen that there is no term of ${\hat{\tau }}_{z}{\hat{\sigma }}_{z}{\hat{s}}_{z}$ in Hamiltonian (1), in comparison with Equation 11 in ref.^26^. Actually, this term is not needed in reproducing the DFT calculated bands from the effective model. It is clear that ${\hat{\tau }}_{z}$ is conserved because of $\left[{\hat{\tau }}_{z},\hat{H}\right]=0$.

We can derive the following energy bands by solving the eigen equation of Hamiltonian (1), $\hat{H}\left(\vec{k}\right)\psi \left(\vec{k}\right)=E\left(\vec{k}\right)\psi \left(\vec{k}\right)$.

(Equation 2) $$\begin{array}{l} E\left(k\right)=\pm \sqrt{\frac{E_{1}\left(k\right)\pm \sqrt{E_{2}\left(k\right)}}{2}}\\ E_{1}\left(k\right)=R^{2}+A^{2}+B^{2}+2v_{F}^{2}k^{2}\\ E_{2}\left(k\right)={\left(R^{2}-A^{2}+B^{2}\right)}^{2}+4v_{F}^{2}\left[R^{2}+{\left(A-B\right)}^{2}\right]k^{2} \end{array}$$

It is clear that both $E_{1}\left(k\right)$ and $E_{2}\left(k\right)$ are always positive and there are four bands (indexed with 0, 1, 2, and 3 from bottom to top).

The parameters $v_{F}$, R, A, and B can be determined by fitting the low-energy bands of the hamiltonian (1) with the DFT-calculated bands. The fitting values are summarized in Table 1. As expected, $v_{F}$ is very large for all the five heterostructures, and the other parameters are very small, with their absolute values being 1.014 meV at most. It is shown in Figure S5 that the bands are isotropic in the neighborhood of the K (K′) point in the Brillouin zone. We compare the spin expectation values $\left(s_{x},s_{y},s_{z}\right)$ of the effective model with those directly calculated from DFT in Figure S6. It is clear that they are in good agreement. It is revealed in Table 1 that the model parameters are not very sensitive to variation of crystal structure. It is also interesting that the symmetric trilayers have R=0 and much smaller A and B.

**Table 1:** The fitting model parameters and the relative total energy values

System	$v_{F}$ (meV · Å)	R (meV)	A (meV)	B (meV)	ΔE (meV)	DRF	QVHE
MoSe_2_/Gr-1	5403.00	−0.349	0.167	0.521	0.000	no	yes
MoSe_2_/Gr-2	5473.10	−0.263	0.213	0.624	−0.066	no	yes
MoSe_2_/Gr-3	5195.41	−0.341	0.217	0.579	−0.089	no	yes
MoSe_2_/Gr-4	5154.35	−0.460	0.151	0.334	−0.123	no	yes
WSe_2_/Gr-1	5430.22	−0.629	0.352	−1.014	0.000	yes	yes
WSe_2_/Gr-2	5366.01	−0.588	0.425	−0.972	−0.002	yes	yes
WSe_2_/Gr-3	5352.73	−0.602	0.471	−0.914	−0.006	yes	yes
WSe_2_/Gr-4	5621.94	−0.654	0.433	−0.958	0.048	yes	yes
MoSe_2_/Gr/WSe_2_	5303.28	−0.329	0.708	−0.514	–	yes	yes
WSe_2_/Gr/WSe_2_	5364.21	0.000	0.090	0.121	–	no	yes
MoSe_2_/Gr/MoSe_2_	5386.36	0.000	0.071	0.061	–	no	yes

The fitting parameters ($v_{F}$, R, A, and B) of the Hamiltonian (1) for the 2D graphene-based heterostructures (and possible structural variants) based on graphene (Gr). The relative total energy (ΔE) values of the structural variants are presented for the bilayer heterostructures, and the features of Dirac-Rashba fermions (DRF) and quantum Valley Hall effect (QVHE) are indicated for all the heterostructures.

### Dirac-Rashba fermion systems

**Figure 3:**
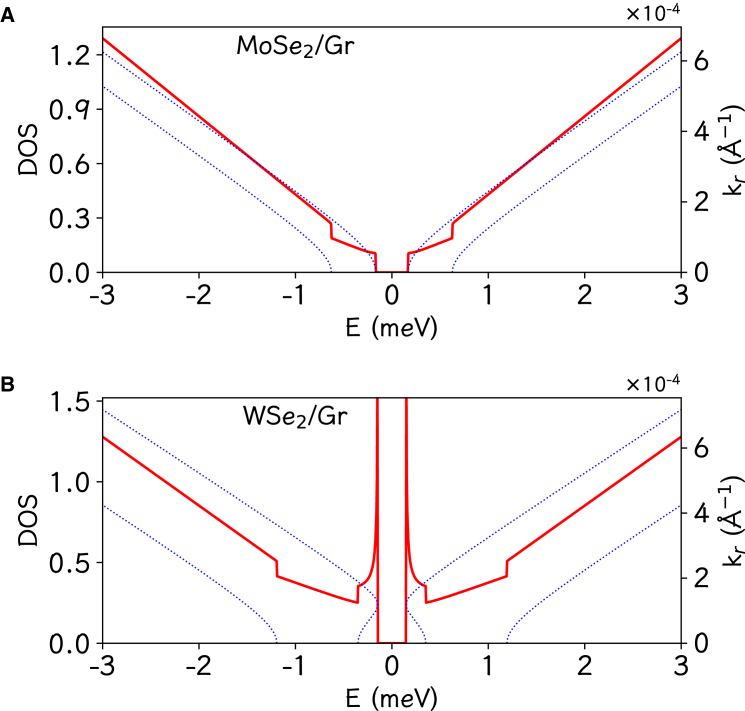
The density of states of representative heterostructures The density of states (DOS, red lines) of the ${\text{MoSe}}_{2}$/graphene (A) and ${\text{WSe}}_{2}$/graphene (B) heterostructures for the corresponding energy bands (blue dots) in the energy window [−3meV, 3meV]. There exists a DOS divergence at both of the band edges in the case of ${\text{WSe}}_{2}$/graphene.

To give more information about the electronic structures, we present in Figure 3 density of states (DOS) with corresponding bands near CBM and VBM in the cases of ${\text{MoSe}}_{2}$/graphene (${\text{MoSe}}_{2}$/Gr-1) and ${\text{WSe}}_{2}$/graphene (${\text{WSe}}_{2}$/Gr-1). For ${\text{MoSe}}_{2}$/graphene, a DOS step is observed at each of the four band edges, which is the feature of two dimensions. In contrast, a big difference appears for ${\text{WSe}}_{2}$/graphene. A divergence of $1/\sqrt{E}$ is observed at both CBM and VBM, in addition to the two DOS steps from the other two bands. Actually, it can be shwon that there will be a circular band edge and then a band-edge divergence of $1/\sqrt{E}$ if the following condition is satisfied.

(Equation 3) $$\left(A^{2}-AB\right)\left(B^{2}+R^{2}-AB\right)>0$$

Such band edges implies that the Rashba effect plays some important role.^29^^,^^31^^,^^33^^,^^52^ Therefore, the effective low-energy model (1) can host Dirac-Rashba fermions because the bands have linear dispersion in the wide windows in addition to the Rashba effect. Their variants have similar DOSs, respectively. The parameters in Table 1 implies that Dirac-Rashba fermions are formed in the ${\text{WSe}}_{2}$/graphene and ${\text{MoSe}}_{2}$/graphene/WSe_2_ (including the variants).

**Figure 4:**
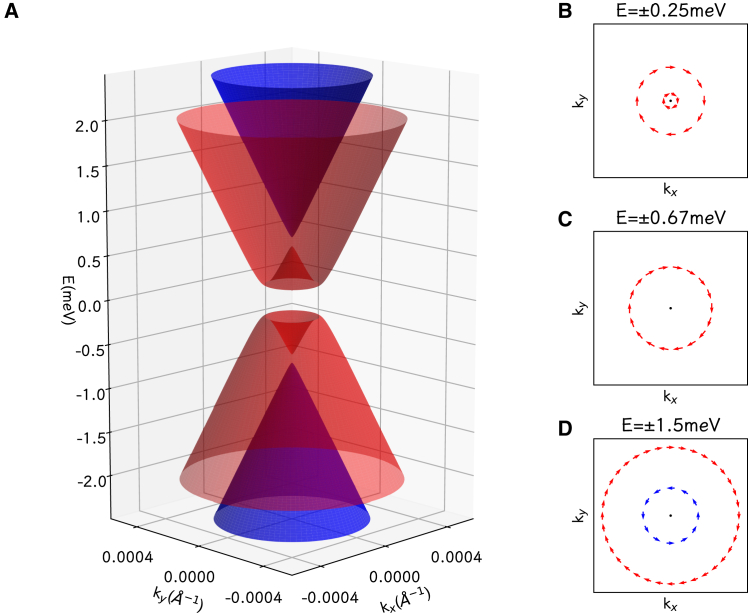
The low-energy electronic bands and spin textures (A) The low-energy electronic bands of the ${\text{WSe}}_{2}$/graphene heterostructure, showing the Dirac-Rashba fermion system, with the zero energy point at the middle of the semiconductor gap. (B–D) The spin textures at energy levels ±0.25 meV (B), ±0.67 meV (C), and ±1.5 meV (D).

**Figure 5:**
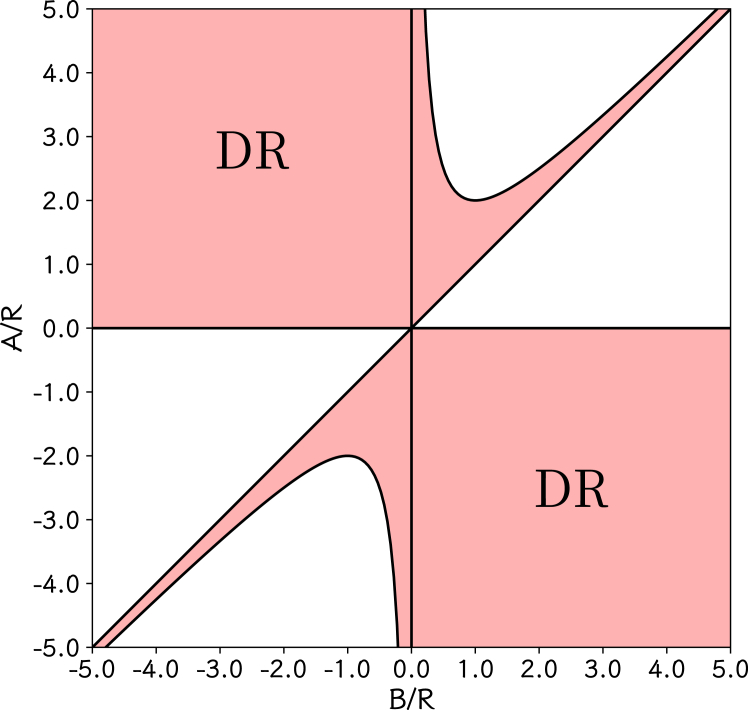
The phase diagram of the Dirac-Rashba fermion systems The phase diagram of the Dirac-Rashba (DR) fermion systems in the parameter space (B/R,A/R).

For the Dirac-Rashba fermions, the energy bands are described by the band structure (2), as shown in Figure 4. There is a symmetry between the valence bands and the conduction bands. The conduction and valence band edges are made by the two circles, and the circular band edges cause a divergence of $1/\sqrt{E}$. It is clear from Table 1 that the parameters ($v_{F}$, R, A, and B) are not sensitive to the inter-layer stacking structures the variants. We plot a phase diagram of (B/R,A/R) for the Dirac-Rashba fermion systems in Figure 5, and present a simple summary of the Dirac-Rashba fermion systems in Table 2.

**Table 2:** The summary of the Dirac-Rashba fermions and quantum valley Hall effect

System	Dirac-Rashba	QVHE
MoSe_2_/Gr	no	yes
WSe_2_/Gr	yes	yes
MoSe_2_/Gr/WSe_2_	yes	yes
WSe_2_/Gr/WSe_2_	no	yes
MoSe_2_/Gr/MoSe_2_	no	yes

The summary of the Dirac-Rashba fermion systems and quantum valley Hall effect (QVHE) in the five 2D heterostructures based on graphene (Gr).

### Quantum valley Hall effect

In order to access topological properties, it is vecessary to calculate the Berry curvature of the energy bands. According to Kubo formula,^53^ the Berry curvature of the n-th band at the point $\vec{k}$, $\Omega^{n}\left(\vec{k}\right)$, can be written as

(Equation 4) $$\Omega^{n}=i\sum_{m\neq n}\frac{\left\langle n\left|{\hat{v}}_{x}\right|m\right\rangle \left\langle m\left|{\hat{v}}_{y}\right|n\right\rangle -\left\langle n\left|{\hat{v}}_{y}\right|m\right\rangle \left\langle m\left|{\hat{v}}_{x}\right|n\right\rangle }{{\left(E_{n}-E_{m}\right)}^{2}}$$

where ${\hat{v}}_{x}=\frac{\partial \hat{H}}{\partial k_{x}}$ and ${\hat{v}}_{y}=\frac{\partial \hat{H}}{\partial k_{y}}$, $E_{n}=E_{n}\left(\vec{k}\right)$ is the n-th band anf $|n\rangle =|n\vec{k}\rangle$ (or $\psi_{n}\left(\vec{k}\right)$) is the corresponding eigen wavefunction. The differential in the formula can be replaced with difference for the numerical calculation. Finally, the value of the Chern number can be obtained by summing the Berry curvature $\Omega^{n}\left(\vec{k}\right)$ in terms of the symmetry of Hamiltonian (1).

There are 4 effective parameters in this hamiltonian model, but three of them are important to the topological properties of this model because the parameter $v_{F}$ only determines the slope of the energy band near the K (K′) point in the Brillouin zone. It is helpful to fix the parameter R and scan the values of A and B in the parameter space to calculate the topological Chern number, and then the topological phase diagram can be plotted with A/R and B/R. The resulting phase diagram of Chern number (C) for the K valley and some band-resolved Chern numbers along two paths are presented in Figures S7 and S8. Here C is the sum of the two valence bands. It is clear in Figure S7 that C=1 in the upper half-plane (A/R>0) and C=−1 in the lower half-plane (A/R<0), which implies that A/R is the key parameter for the Chern number. It is easy to show that the semiconductor gap (at the Fermi level) is closed along the line A=0. The three points T1, T2, and T3 correspond to the three heterostructures: ${\text{MoSe}}_{2}$/graphene/WSe_2_, ${\text{MoSe}}_{2}$/graphene, and ${\text{WSe}}_{2}$/graphene. It should be pointed out that all the C values are reversed for the K′ valley because of time reversal symmetry.

We can use ${\hat{\tau }}_{z}$ and the corresponding xy components to define a pseudo-spin operator for the valley degree of freedom, $\hat{\vec{\eta }}=\left(\hbar /2\right)\hat{\vec{\tau }}$, and thus ${\hat{\eta }}_{z}$ is also conserved. It is similar to the z component of the real spin operator. $\eta_{z}=\hbar /2$ for the K valley, and $\eta_{z}=-\hbar /2$ for the K′ valley. Consequently, we obtain $C_{\eta }=\left(C_{K}-C_{K^{\prime }}\right)/2=1$ for A/R>0 and $C_{\eta }=\left(C_{K}-C_{K^{\prime }}\right)/2=-1$ for A/R<0. It should be pointed out that $C_{\eta }=1$ (mod 2) for |A/R|>0 and $C_{\eta }=0$ along the line A=0. Therefore, the model (1) can host a quantum valley Hall effect, similar to quantum spin Hall effect.^23^^,^^24^ This is different from another quantum valley Hall effect achieved without Berry curvature.^54^ It is also different from interesting ferrovalley effect.^55^^,^^56^^,^^57^^,^^58^^,^^59^ Here, the Rashba term makes the band edge become a circle (1D) and plays vital role. As shown in Figure S7, ${\text{MoSe}}_{2}$/graphene/WSe_2_, ${\text{MoSe}}_{2}$/graphene, and ${\text{WSe}}_{2}$/graphene are in the region determined by A/R<0 and have $C_{\eta }=-1$. They can be considered to be quantum valley Hall insulators. Because the parameters ($v_{F}$, R, A, and B) are not sensitive to different inter-layer stacking structures, as shown in Table 1, we also make a simple summary for our results of quantum valley Hall effect in the graphene-based heterostructures in Table 2.

### Further discussion

The phase diagram can be divided into four regions (I, II, III, and IV) by means of the curves ${\left(A/R\right)}^{2}-{\left(B/R\right)}^{2}=1$ and A/R=0. The two paths are used to show the band-resolved Chern numbers, as presented in Figure S8. The two valence bands (bands 0 and 1) have different contribution to the Chern number C, and one is larger than C/2 and the other smaller than C/2. It is interesting that the increments are opposite with respect to C/2 and will exchange sign when the curve ${\left(A/R\right)}^{2}-{\left(B/R\right)}^{2}=1$ is crossed. It can be shown from the band expression (2) that the two valence bands at $\vec{k}=0$ (K, K′) become degenerate when ${\left(A/R\right)}^{2}-{\left(B/R\right)}^{2}=1$ is satisfied. When |R| is small, A/R and B/R can be out of the parameter region in the phase diagram. Fortunately, we have shown that the phase diagram can be extended to large A/R and B/R (to ±∞).

Furthermore, it is interesting to explore the interaction between the orbitals and the spins, and elucidate their relationships with the Berry curvature. We can calculate band-resolved average values of $\left({\hat{\sigma }}_{x},{\hat{\sigma }}_{y},{\hat{\sigma }}_{z}\right)$ and $\left({\hat{s}}_{x},{\hat{s}}_{y},{\hat{s}}_{z}\right)$ between the eigen-functions $|n\rangle =|n\vec{k}\rangle$. The average values of orbitals and spins can be written as

(Equation 5) $${\vec{\sigma }}^{n}\left(\vec{k}\right)=\left\langle n\vec{k}\left|\hat{\vec{\sigma }}\right|n\vec{k}\right\rangle ,{\vec{s}}^{n}\left(\vec{k}\right)=\left\langle n\vec{k}\left|\hat{\vec{s}}\right|n\vec{k}\right\rangle$$

**Figure 6:**
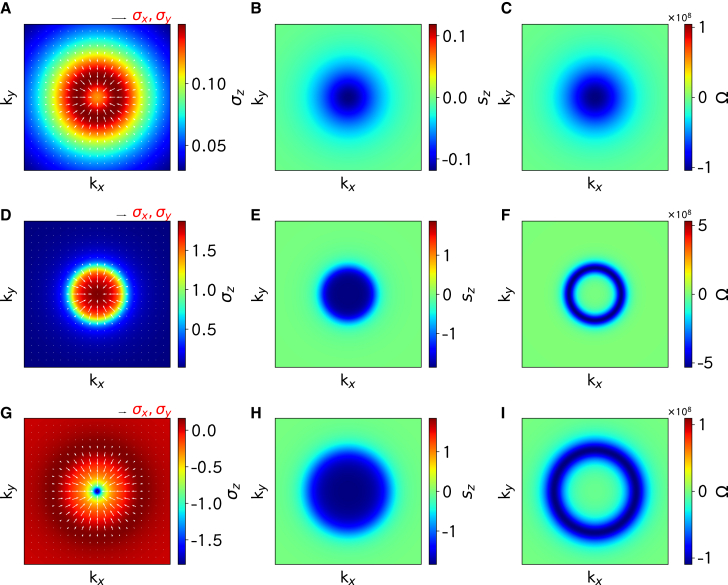
Schematic k-space distributions of orbital textures, spin textures, and Berry curvatures of the two valence bands Schematic k-space distributions of orbital textures ($\Delta \vec{\sigma }$ =($\sigma_{x}^{0}$-$\sigma_{x}^{1},\sigma_{y}^{0}$-$\sigma_{y}^{1},\sigma_{z}^{0}$-$\sigma_{z}^{1}$), left column), spin textures ($s_{z}$ = $s_{z}^{0}$ + $s_{z}^{1}$, middle column), and Berry curvatures (Ω = $\Omega^{0}$ + $\Omega^{1}$, right column) of the two valence bands (0 and 1) for ${\text{MoSe}}_{2}$/graphene (A–C), ${\text{WSe}}_{2}$/graphene (D–F) and ${\text{MoSe}}_{2}$/graphene/WSe_2_ (G–I) as 2D heterostructures. The white arrows describe the direction and size of the xy components ($\Delta \sigma_{x},\Delta \sigma_{y}$) (A, D, and G), and the color scales are used to indicate the $\Delta \sigma_{z}$ (A, D, and G), $s_{z}$ (B, E, and H), and Ω (C, F, and I), respectively.

It is expected that ${\vec{\sigma }}^{n}\left(\vec{k}\right)$ and ${\vec{s}}^{n}\left(\vec{k}\right)$ will converge to those of monolayer graphene when the k-vector is far from the K (K′) point, and the main difference will limited to the small regions near K (K′) point (except some band translation), as shown in Figure S9. While the three band-resolved average values ${\vec{\sigma }}^{n}\left(\vec{k}\right)$ are nonzero, $\Delta \vec{\sigma }\left(\vec{k}\right)=\left(\sigma_{x}^{0}-\sigma_{x}^{1},\sigma_{y}^{0}-\sigma_{y}^{1},\sigma_{z}^{0}-\sigma_{z}^{1}\right)$ is nonzero only near the K (K′) point. It is interesting that the xy components $\left(\Delta \sigma_{x},\Delta \sigma_{y}\right)$ remains in the radial direction in the xy plane. There are band-resolved helical spin textures ${\vec{s}}^{n}\left(\vec{k}\right)$ due to the Rashba term, but the horizontal components ($s_{x}=s_{x}^{0}+s_{x}^{1}$ and $s_{y}=s_{y}^{0}+s_{y}^{1}$) of the two valence bands are equivalent to zero because the two valence bands have opposite contributions to the xy spin components, and then only the z component $s_{z}=s_{z}^{0}+s_{z}^{1}$ can be nonzero. To effectively show the relationship between the orbitals and the spins, we present in Figure 6 $\Delta \vec{\sigma }$ and $s_{z}$ of the ${\text{MoSe}}_{2}$/graphene, ${\text{WSe}}_{2}$/graphene, and ${\text{MoSe}}_{2}$/graphene/WSe_2_. We focus on $\Delta \vec{\sigma }\left(\vec{k}\right)$ and $s_{z}\left(\vec{k}\right)$ because they are nonzero only near the K (K′) point in the Brillouin zone. These correlated distributions near the K (K′) point reflect the interaction between the orbitals and the spins.

We calculate band-resolved Berry curvature $\Omega^{n}\left(\vec{k}\right)$ of the 2D heterostructures and show those for the ${\text{MoSe}}_{2}$/graphene, ${\text{WSe}}_{2}$/graphene, and ${\text{MoSe}}_{2}$/graphene/WSe_2_ in Figure S9. We also present Berry curvature $\Omega \left(\vec{k}\right)=\Omega^{0}\left(\vec{k}\right)+\Omega^{1}\left(\vec{k}\right)$ near the K (K′) point in Figure 6. It is very interesting that the Berry curvature has main contribution near the band edges (the VBMs) and decays fast and diminishes to zero when the k-vector becomes far from the K (K′) point. For the ${\text{MoSe}}_{2}$/graphene, $\Omega \left(\vec{k}\right)$ reaches its maximum at the K (K′) point. In contrast, for the ${\text{WSe}}_{2}$/graphene and the ${\text{MoSe}}_{2}$/graphene/WSe_2_, $\Omega \left(\vec{k}\right)$ takes its maximum along the circle around the K (K′) point in the Brillouin zone (defined by the valence band edge due to the Rashba effect).

### Conclusion

In summary, five 2D heterostructures consisting of graphene and monolayer TMDs are investigated by means of first-principles investigation. We choose ${\text{MoSe}}_{2}$ and ${\text{WSe}}_{2}$ to align the Dirac cones of graphene with the intrinsic Fermi levels of the TMDs. It is found that the Dirac energy bands of graphene are modified (gaps: 0.1 ∼ 0.5 meV) by the TMDs, and the linear band dispersion near the K and K′ points is kept in a wide energy window of [−0.2 eV, 0.2 eV] at least. We use an effective low-energy hamiltonian model to describe the energy bands and analyze the electronic properties, including the topological feature. It is revealed that the model parameters of the effective hamiltonian can be determined by fitting the first-principles energy bands for each of the 2D heterostructures. It is shown that the effective low-energy model hosts Dirac-Rashba fermions in the ${\text{WSe}}_{2}$/graphene and ${\text{MoSe}}_{2}$/graphene/WSe_2_, and there is quantum valley Hall effect in all the graphene-based 2D heterostructures (nearly independent of stacking variation). Our further analyses indicate that the orbitals and valleys can be described by the corresponding pseudo-spins, and there are strong interactions between the orbitals and spins especially near the K and K′ points. These can be useful for exploring more properties and functionalities in 2D materials and 2D heterostructures for promising devices.

### Limitations of the study

This study was performed without considering any moiré pattern of heterostructures. It is based on experimental observation that the moiré pattern of a 2D heterostructure can be eliminated by annealing process at a high temperature and high stability can be achieved in 2D heterostructures without moiré pattern.^22^

## Resource availability

### Lead contact

Requests for further information and resources should be directed to and will be fulfilled by the lead contact, Bang-Gui Liu (bgliu@iphy.ac.cn).

### Materials availability

This study did not generate new materials.

### Data and code availability

• All data reported in this paper will be shared by the lead contact upon request.
• VASP is used for the first-principles calculation and analysis concerned and can be accessed at https://www.vasp.at.
• The figures are plotted with Matplotlib (https://matplotlib.org/) in terms of the first-principles calculated data.

## Acknowledgments

This work is supported by the 10.13039/501100001809National Natural Science Foundation of China (grant no.11974393) and the Strategic Priority Research Program of the 10.13039/501100002367Chinese Academy of Sciences (grant no. XDB33020100).

## Author contributions

Conceptualization, B.-G.L.; methodology, B.-G.L. and B.-W.Y.; investigation, B.-W.Y.; writing – original draft, B.-W.Y.; writing – review and editing, B.-G.L.; funding acquisition, B.-G.L.; resources, B.-G.L.; supervision, B.-G.L.

## Declaration of interests

The authors declare no competing interests.

## STAR★Methods

### Key resources table

**Table:** 

REAGENT or RESOURCE	SOURCE	IDENTIFIER
**Software and algorithms**		

VASP code	VASP Software GmbH	https://www.vasp.at/
Graph plotting	Matplotlib	The Matplotlib development team (https://matplotlib.org/)

### Experimental model and study participant details

No experimental model and participant in this study.

### Method details

The first-principles calculations are performed with the projector-augmented wave (PAW) method within the density functional theory,^60^ as implemented in the Vienna Ab-initio simulation package software (VASP).^61^ The generalized gradient approximation (GGA) by Perdew, Burke, and Ernzerhof (PBEs)^62^ is used as the exchange-correlation functional. Our computational supercell includes 4×4 primitive cells of monolayer graphene and 3×3 primitive cells of H-TMD monolayers (the detailed structures and parameters will be presented in the following). The self-consistent calculations are carried out with a Γ-centered (4×4×1) Monkhorst-Pack grid of the supercell.^63^ The kinetic energy cutoff of the plane wave is set to 450 eV. The convergence criteria of the total energy and force are set to $10^{-6}$ eV and 0.01 eV/Å. A test is done with graphene-based heterostructure ${\text{MoSe}}_{2}$/graphene for better convergence criteria of force (0.007 and 0.0035 eV/Å), with the total energy differences (0.00 and -0.18 meV per unit cell), and no differences can be seen in the electronic bands plotted in Figure S1. The spin-orbit coupling (SOC) is taken into account in the calculation of band structures and optimization of lattice structures. The inter-layer vacuum thickness is set to at least 30 Å. The dipole correction^64^^,^^65^ is included in the calculation for the heterostructures. Dispersion corrections are taken into account via the Grimme approximation (DFT-D3).^66^

### Quantification and statistical analysis

The data was produced by using VASP, providing enough precision and having no dependence on parameters. There is no statistical analysis because this study is based on first-principles calculations.
