# Supplementary material for: Dirac-Rashba fermions and quantum valley Hall insulators in graphene-based 2D heterostructures
Source: iScience. 2025 Jun 4;28(7):112818. doi: 10.1016/j.isci.2025.112818 (PMC12221682; doi:10.1016/j.isci.2025.112818)
Supplement: Document S1. Figures S1–S9 [file mmc1.pdf]

iScience, Volume *28*

Supplemental information

Dirac-Rashba fermions and quantum valley Hall insulators in graphene-based 2D heterostructures

Bo-Wen Yu and Bang-Gui Liu

Supplemental Materials for

Dirac-Rashba fermions and quantum valley Hall insulators in graphene-based 2D heterostructures

Bo-Wen Yu and Bang-Gui Liu

Beijing National Laboratory for Condensed Matter Physics, Institute of Physics, Chinese Academy of Sciences, Beijing 100190, China

School of Physical Sciences, University of Chinese Academy of Sciences, Beijing 100049, China

Email : bgliu@iphy.ac.cn,   OrcidID: 0000-0002-6030-6680

Here, we present more detailed information:

- Confirmation of the convergence criteria of force (Fig. S1),
- The top view of the structural variants of the bilayer heterostructures (Fig. S2),
- The crystal structures of the trilayer heterostructures (Fig. S3),
- Electronic structures of the other four heterostructures (Fig. S4),
- Showing isotropic property of the band structures near the K point (Fig. S5),
- The spin textures near the K point in the K-M direction (Fig. S6),
- The topological phase diagram for the K valley (Fig. S7),
- The band-resolved and total Chern numbers along two paths in Fig. S7 (Fig. S8),
- Schematic band-resolved k-space distributions of the energy dispersions, orbital textures, spin textures, and Berry curvatures (Fig. S9).

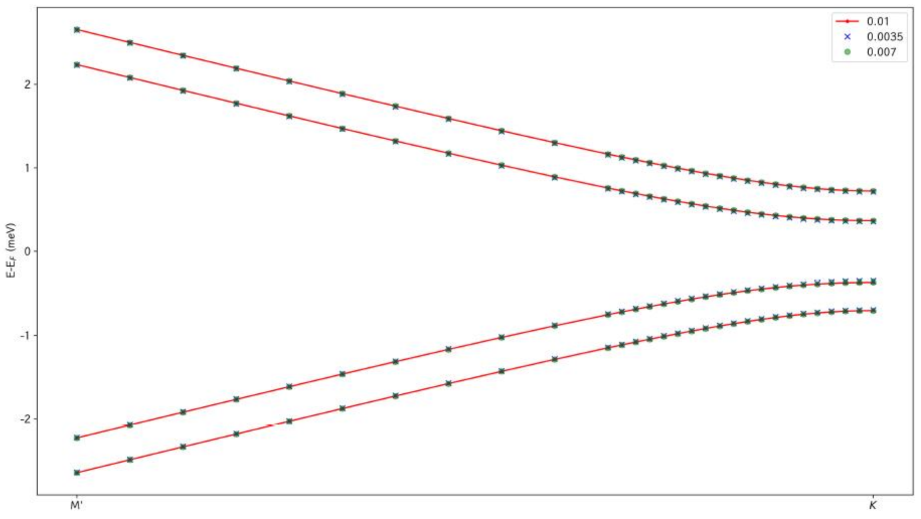

Fig. S1. Confirmation of the convergence criteria of force. Comparison of the energy bands of the bilayer heterostructure MoSe2/Graphene for different convergence criteria of force: 0.01, 0.007, and 0.0035 eV/Å.

.

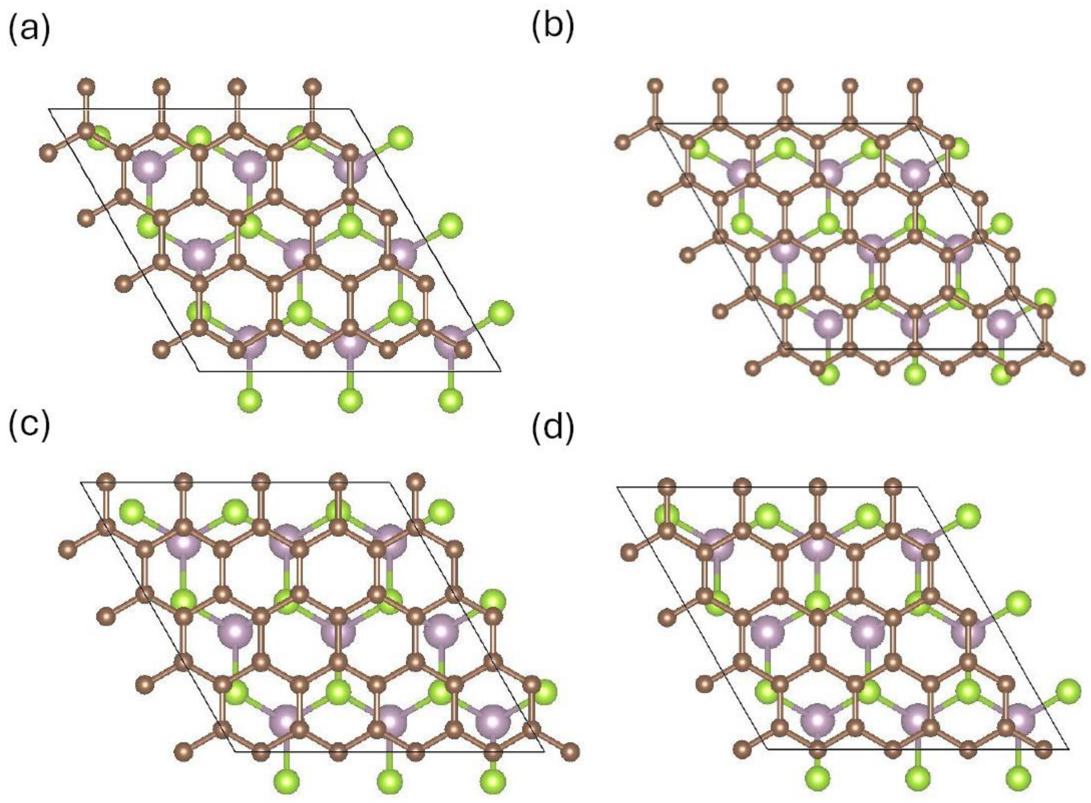

Fig. S2. The top view of the structural variants of the bilayer heterostructures. (a) The top view of structure XSe_2/Gr-1; (b) The top view of structure XSe_2/Gr-2; (c) The top view of structure XSe_2/Gr-3; and (d) The top view of structure XSe_2/Gr-4. Here, X denotes Mo or W, and XSe_2/Gr-*i* is the *i*-th structural variant of XSe_2/Gr ($MoSe_2$/graphene or WSe_2/graphene).

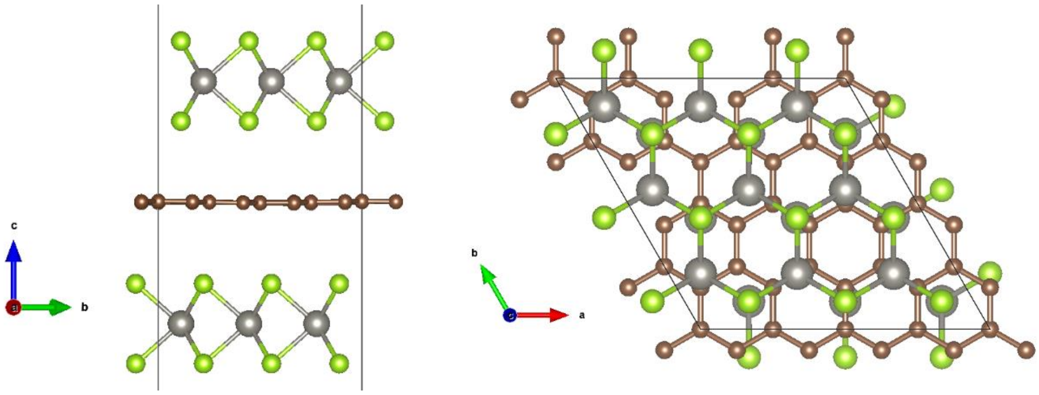

Fig. S3. The crystal structures of the trilayer heterostructures. The side view (left) and top view (right) of the structure of the three-monolayer model of the heterostructure used.

(A) $MoSe_2$/graphene

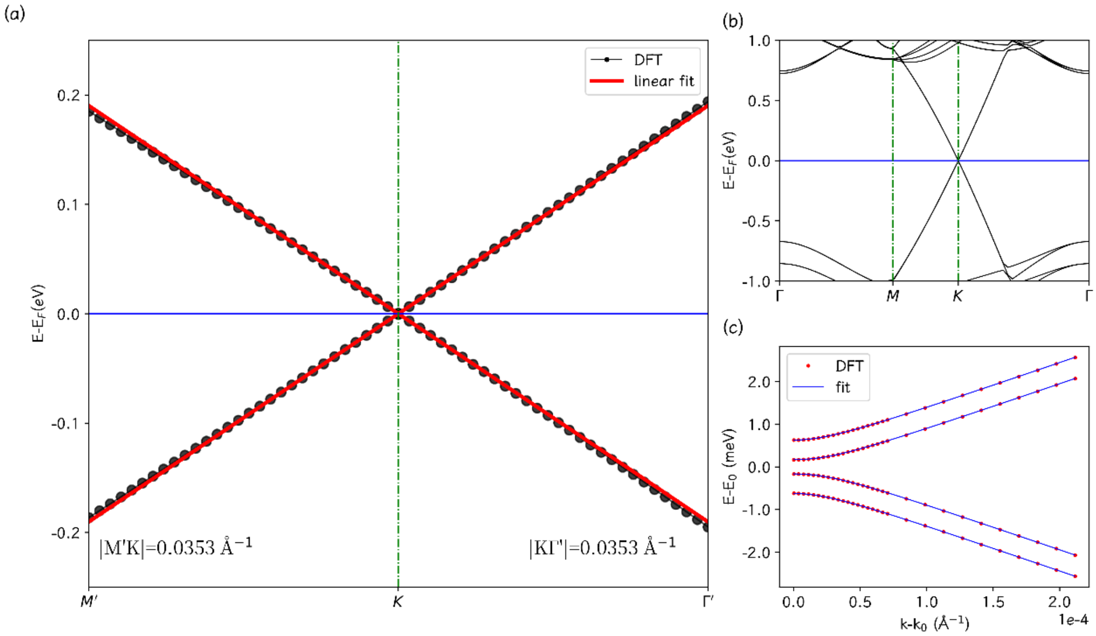

(B) WSe_2/graphene

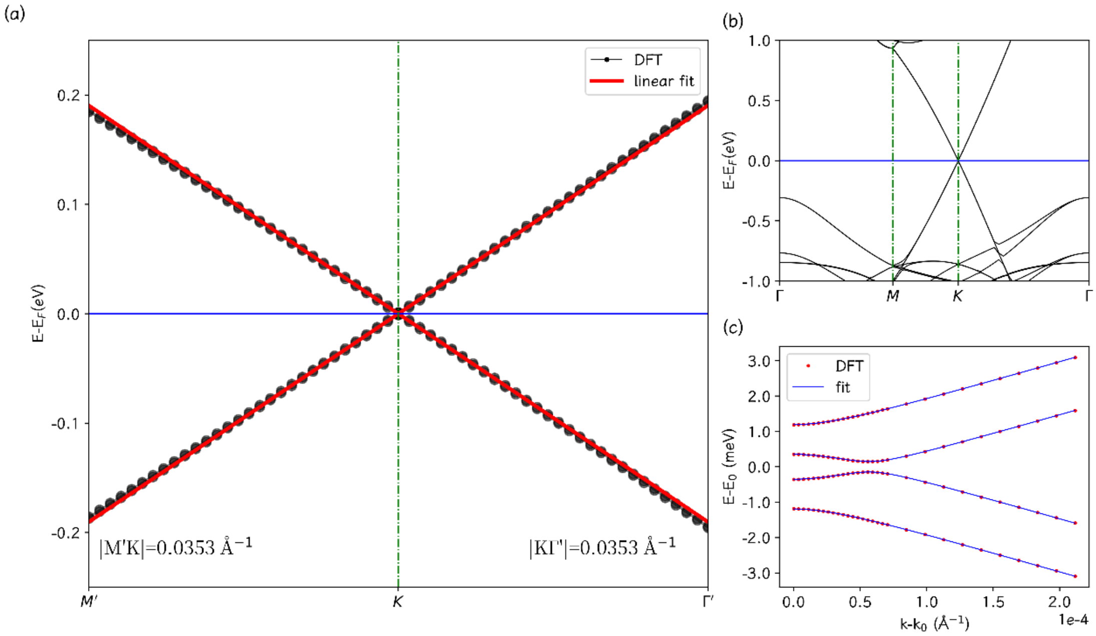

(C) MoSe2/graphene/MoSe2

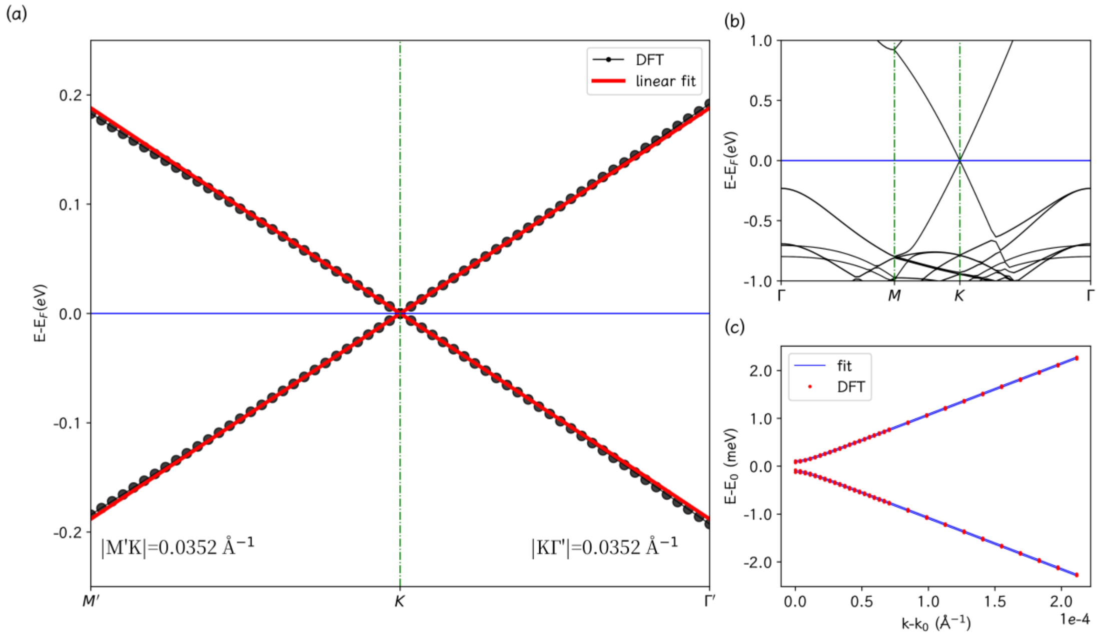

(D) WSe2/graphene/WSe2

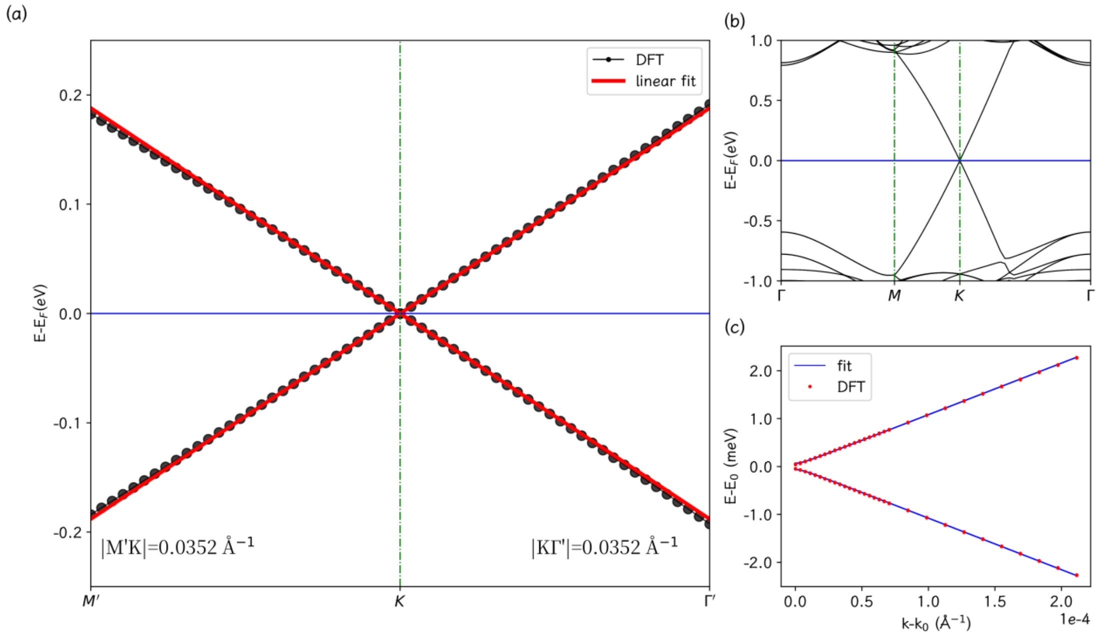

Fig. S4. Electronic band structures of the other four heterostructures. (A) $MoSe_2$/graphene, (B) WSe_2/graphene, (C) $MoSe_2$/graphene/$MoSe_2$, and (D) $MoSe_2$/graphene/$MoSe_2$. There are three panels (a, b, c) in each case. (a) the bands near K point in a relatively large range of Brillouin zone. The distance between the M' or Γ and K point is 0.0352 Å^{-1}. The black line is the DFT-calculated results and the red line is the linear fitting, which indicates the broad linear range. (b) The band structure near the Fermi level in the range of whole Brillouin zone. It shows that the composite heterostructure contains the features from both the graphene and the 2D TMDs and the Dirac cones are approximately at the middle of the semiconductor gaps of the TMDs. (c) The band structure near the Fermi level near the K point in a very small range.

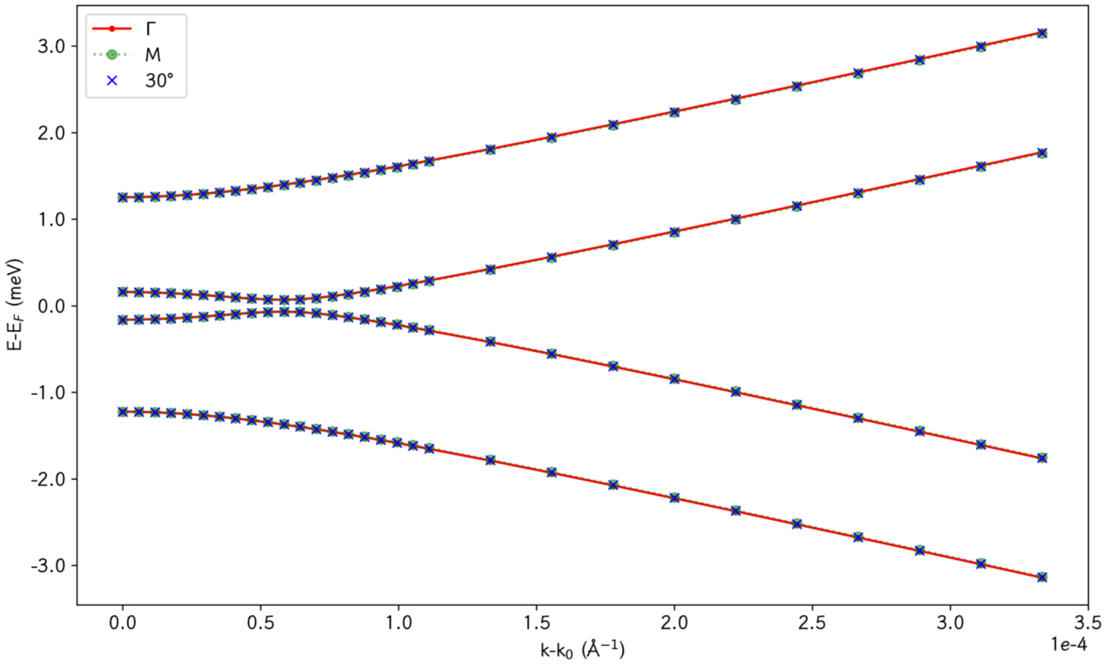

Fig. S5. Showing isotropic property of the band structures near the K point. The band structure of the 2D heterostructure near the K point in a very small range in different directions. The red line is the band along the K-Γ direction, the green line is the band along the K-M direction and the blue line is the band along the direction with an angle of 30 degrees to K-M. The results show that the energy band for the three directions is the same and there is no anisotropy, which proves the rationality of removing the anisotropic term from the Hamiltonian.

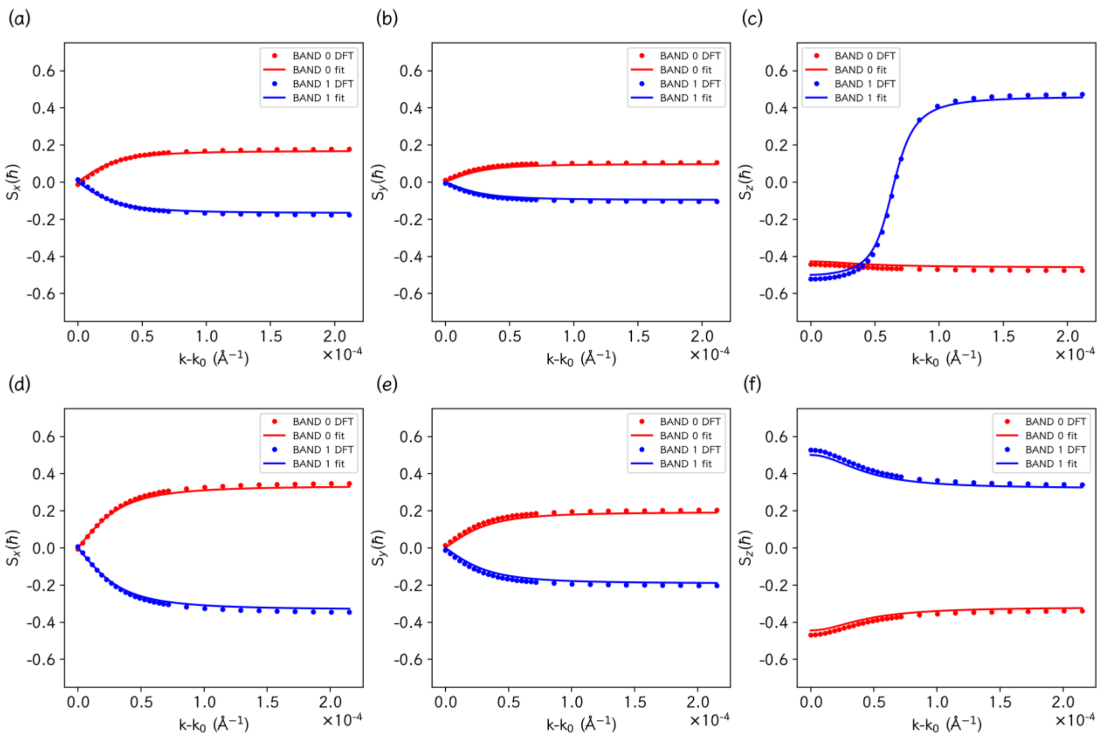

Fig. S6. The spin textures near the K point in the K-M direction. The s_x, s_y and s_z spin expectation values near the K point in the K-M direction from the DFT calculation (dots) and the effective model (solid lines) for WSe_2/graphene heterostructure (a,b,c) and $MoSe_2$/graphene heterostructure (d,e,f). The weights of the orbitals (projection) near the K point are 0.525 for p orbitals and 0.002 for d orbitals in the WSe_2/graphene, and 0.521 for p orbitals and 0.002 for d orbitals in the $MoSe_2$/graphene.

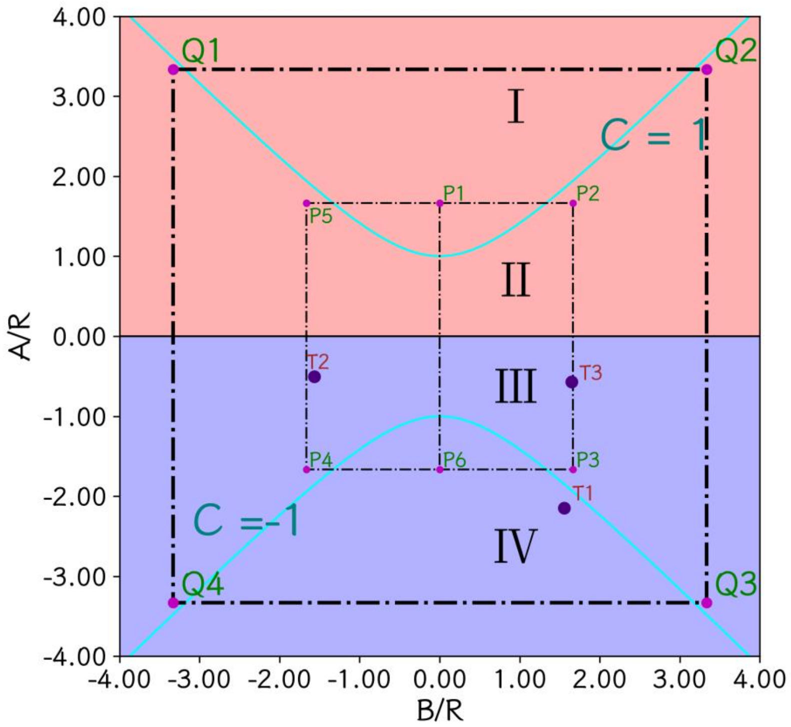

Fig. S7. The topological phase diagram for the K valley. The Chern number for the K valley, *C*, is 1 (-1) in the upper (lower) half. The cyan line is defined by $(A/R)^2$-$(B/R)^2$ =1. The point T1 is for the $MoSe_2$/graphene/WSe_2 heterostructure, T2 for the $MoSe_2$/graphene, T3 for the WSe_2/graphene.

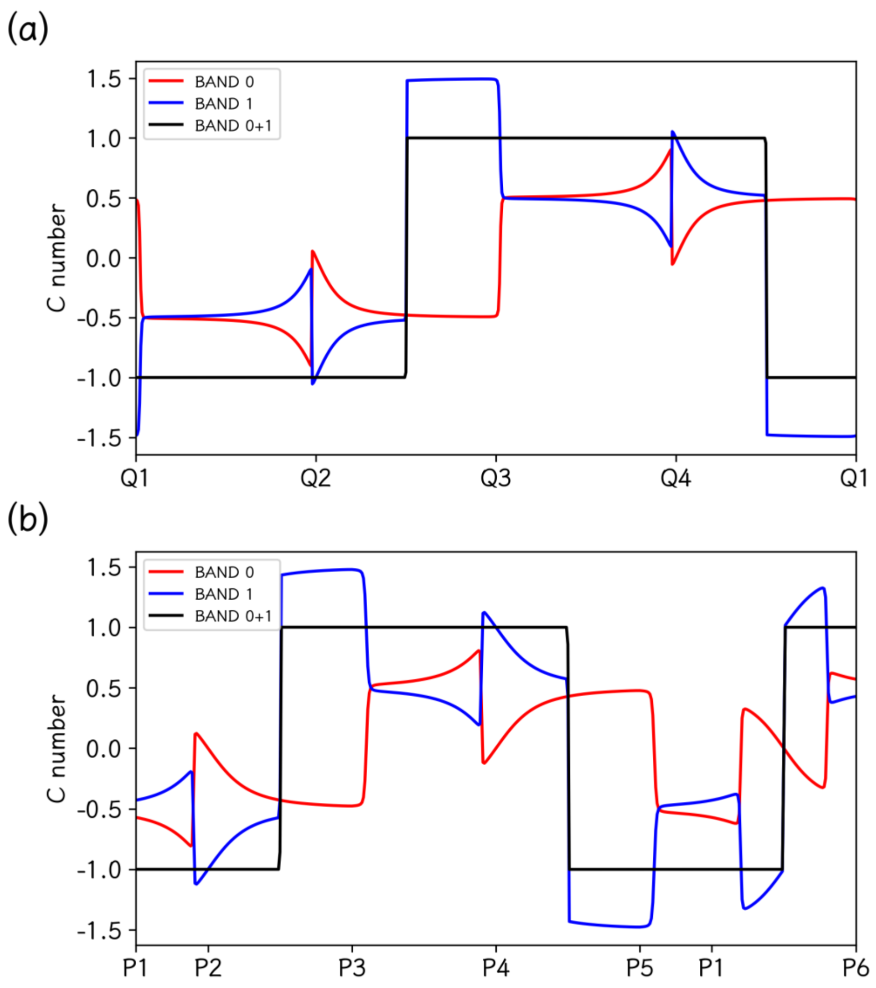

Fig. S8. The band-resolved and total Chern numbers along two paths in Fig. S7. (a) The band-resolved (red/blue) and the total (black) Chern numbers along path Q1-Q2-Q3-Q4-Q5-Q1 in the phase diagram shown in Fig. S7. (b) The Chern numbers along P1-P2-P3-P4-P5-P1-P6 in Fig. S7. The red (blue) lines are for the lower (upper) valence band.

(A) $MoSe_2$/Gr

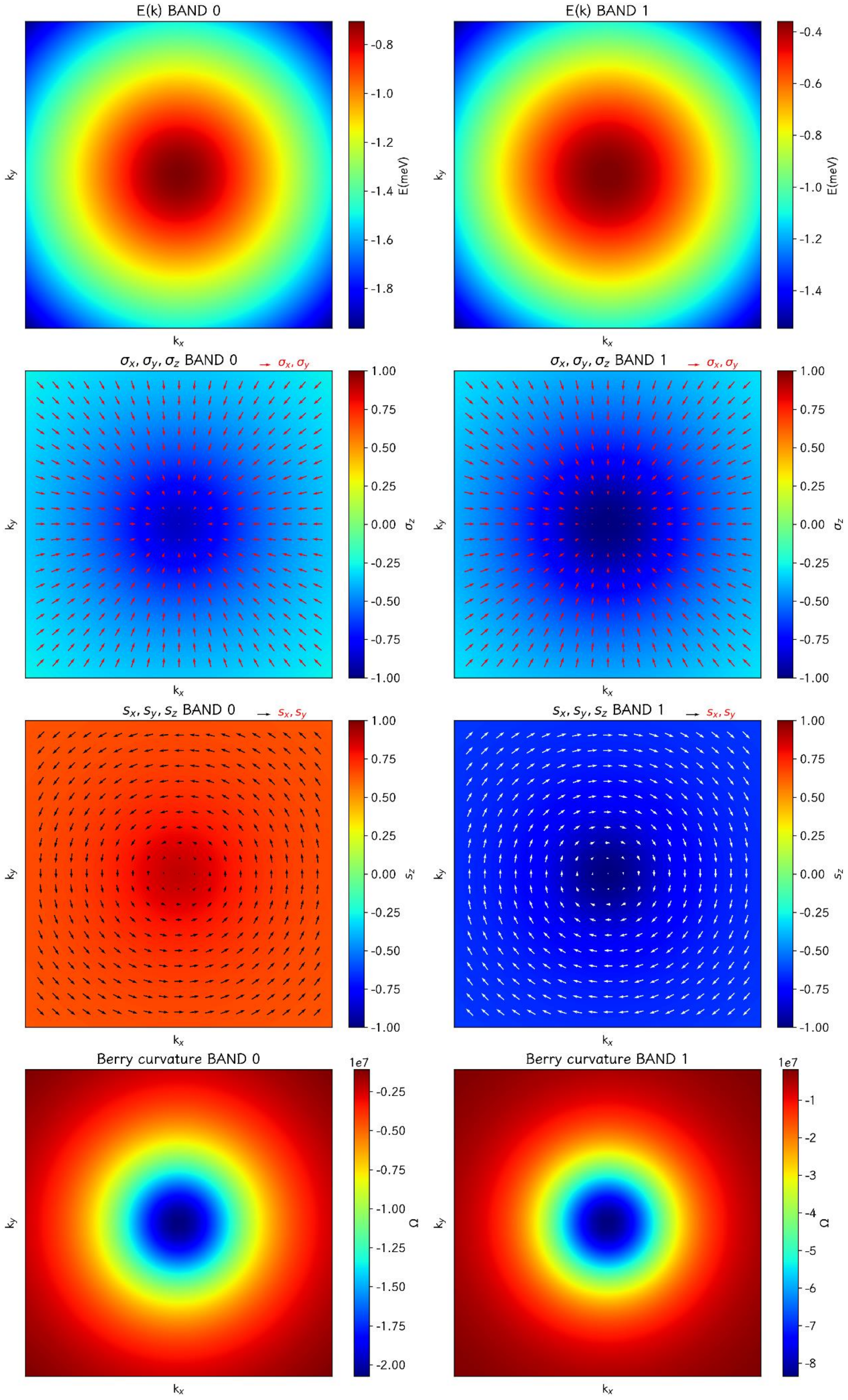

E(k) BAND 0
E(k) BAND 1
E(meV)
$\sigma_x, \sigma_y, \sigma_z$ BAND 0
$\sigma_x, \sigma_y, \sigma_z$ BAND 1
σ_x, σ_y
σ_z
s_x, s_y, s_z BAND 0
s_x, s_y, s_z BAND 1
s_x, s_y
s_z
Berry curvature BAND 0
Berry curvature BAND 1
1e7
Ω
k_x
k_y

(B) WSe_2/Gr

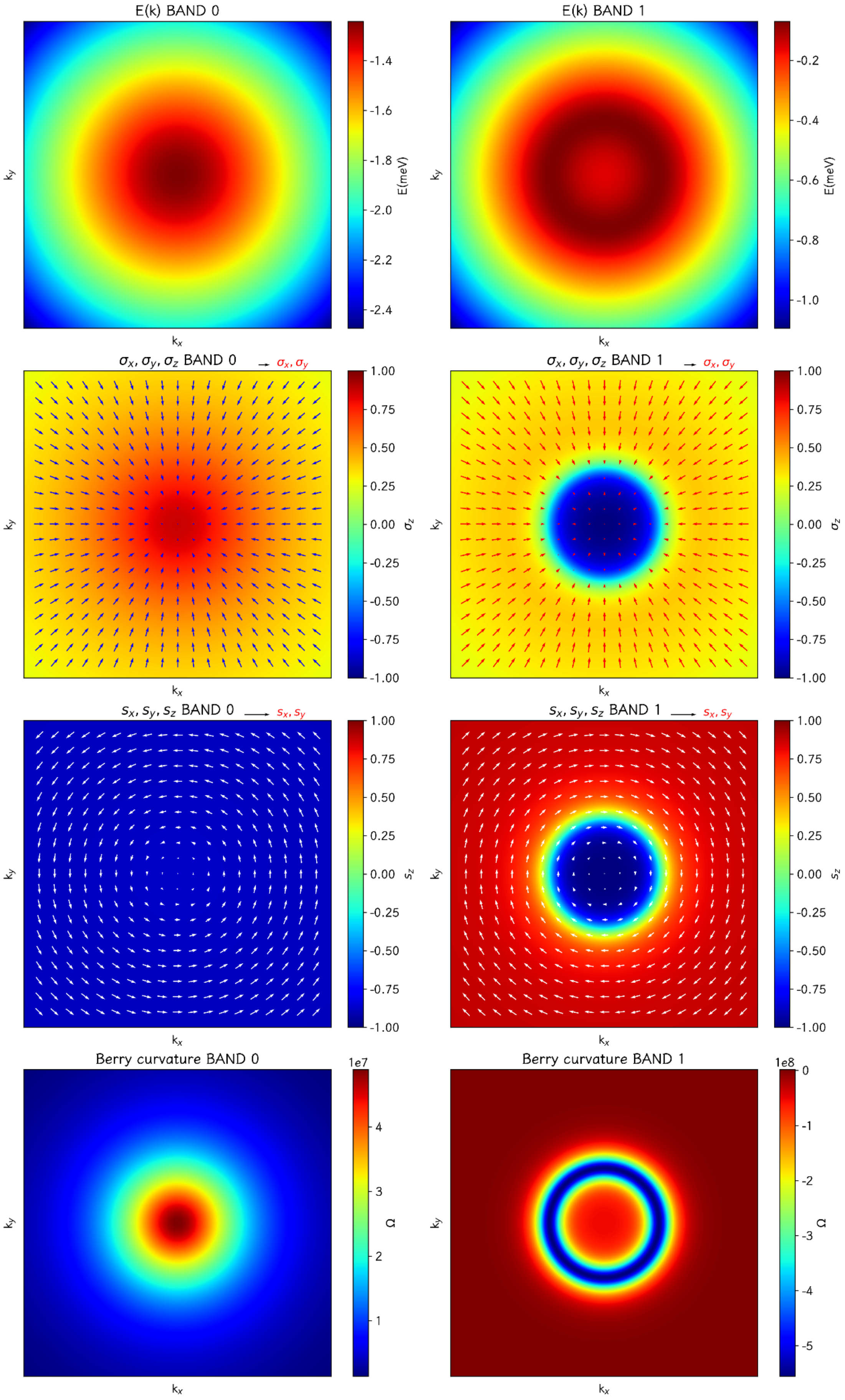

E(k) BAND 0
E(k) BAND 1
E(meV)
σx, σy, σz BAND 0
σx, σy, σz BAND 1
σx, σy
σz
sx, sy, sz BAND 0
sx, sy, sz BAND 1
sx, sy
sz
Berry curvature BAND 0
Berry curvature BAND 1
1e7
1e8
Ω
kx
ky

(C) $MoSe_2/Gr/WSe_2$

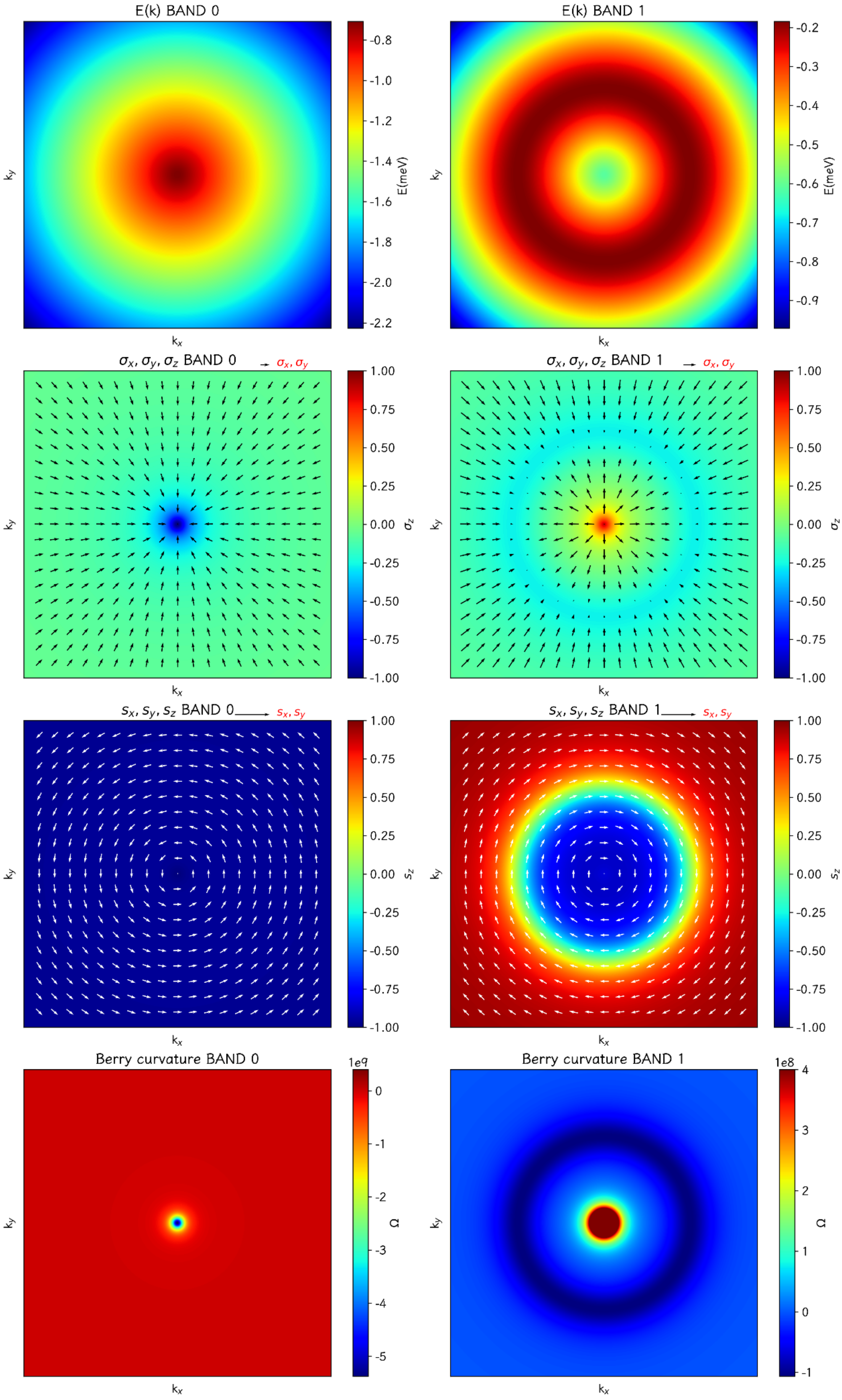

Fig. S9. Schematic band-resolved k-space distributions of the energy dispersions, orbital textures, spin textures, and Berry curvatures. Band-resolved k-space distributions of the energy dispersion relation $E^n(k_x, k_y)$, orbital textures $(\sigma^n{}_x, \sigma^n{}_y, \sigma^n{}_z)$, spin textures $(s^n{}_x, s^n{}_y, s^n{}_z)$ and Berry curvatures (Ω^n) of the two valence bands (n=0,1) are presented for three heterostructures. (A) $MoSe_2$/graphene, (B) WSe_2/graphene, and (C) $MoSe_2$/graphene/WSe_2. In each of the three cases, the arrow indicate the direction and size of the value of the vector $(\sigma^n{}_x, \sigma^n{}_y)$ or $(s^n{}_x, s^n{}_y)$ and the corresponding color scales are used to indicate $E(k_x, k_y)$, $\sigma^n{}_z$, $s^n{}_z$, and Ω^n respectively. The superscript n can be hidden for brevity, as is done in the figures.
